# Comparative study of conventional steam cooking and microwave cooking on cooked pigmented rice texture and their phenolic antioxidant

**DOI:** 10.1002/fsn3.1377

**Published:** 2020-01-09

**Authors:** Sukanya Thuengtung, Yukiharu Ogawa

**Affiliations:** ^1^ Graduate School of Horticulture Chiba University Matsudo Japan

**Keywords:** anthocyanins, cooking process, phenolic antioxidant, pigmented rice, rice texture

## Abstract

The impact of two different cooking processes (microwave and steaming) on cooked rice quality (i.e., texture), and changes in the bioactive compounds (total phenolic content [TPC] and total anthocyanin content [TAC]) and antioxidant activities (DPPH and FRAP assays) of black and red (nonwaxy) and purple (waxy) pigmented rice were investigated. No significant difference in the firmness between microwave‐cooked rice and steam‐cooked rice was found, except for cooked purple rice. However, microwave cooking promoted an increase in the cooked rice adhesiveness, which approximately higher 2‐ ~ 3‐fold than that of steam cooking with varying among rice cultivars. Microwave cooking also exhibited significantly higher TPC (1.2‐ ~ 2.0‐fold), TAC (2.0‐ ~ 3.2‐fold), DPPH (1.3‐ ~ 2.5‐fold), and FRAP (1.5‐ ~ 2.4‐fold) than steam cooking for black and purple rice cultivars. There was a strong positive correlation among these bioactive compounds and the antioxidant activities (*p* < .01). Our study indicated that the TPC, TAC, DPPH, and FRAP of all rice examined were remarkably decreased after cooking, and the extent of the decrease depended on the rice cultivar and cooking method.

## Graphical abstract

The bioactive compounds such as TPC, TAC, and antioxidant activities like DPPH and FRAP of pigmented rice were remarkably decreased after cooking, and the extent of the decrease depended on the rice cultivar and cooking method.

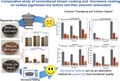

## 1 INTRODUCTION

Pigmented rice can be characterized into groups of black, purple, and red rice, distinguished by the color of the pericarp layer of the rice grain (Sompong, Siebenhandl‐Ehn, Linsberger‐Martin, & Berghofer, 2011). Several bioactive compounds exist in pigmented rice, notably phenolic compounds, which possess numerous health benefits, such as antioxidant and anti‐inflammatory effects, and reduce the risk of chronic diseases (Bhawamai, Lin, Hou, & Chen, 2016; Chatthongpisut, Schwartz, & Yongsawatdigul, 2015; Zhang et al., 2015).

Phenolic compounds are highly diverse, with over 10,000 known phenolic structures, and are generally classified according to the number of phenol rings (Thuengtung & Ogawa, 2019). These molecules are secondary metabolites of plants that possess one or more aromatic rings connected by one or more hydroxyl groups (Dai & Mumper, 2010). Anthocyanins, a group of flavonoids, are the major phenolic compounds found in pigmented rice and are located in the aleurone layer (Walter & Marchesan, 2011). Anthocyanins are water‐soluble compounds that can be easily lost during processing (Norkaew et al., 2017; Patras, Brunton, O'Donnell, & Tiwari, 2010; Siah, Wood, Agboola, Konczak, & Blanchard, 2014). In particular, thermal processes, such as cooking, drying, blanching, and pasteurization, have a major impact on the loss of anthocyanins and other phenolic compounds (Hiemori, Koh, & Mitchell, 2009; Patras et al., 2010). Several studies have shown that the high heating temperatures used during cooking influence the degradation of anthocyanins and other phenolic compounds in pigmented rice, as well as their antioxidant capacities (Bhawamai et al., 2016; Surh & Koh, 2014; Zaupa, Calani, Del Rio, Brighenti, & Pellegrini, 2015). Additionally, some phenolic compounds could be lost from the food materials through the soaking process and then further degraded during cooking (Siah et al., 2014).

Rice is generally consumed as a completely cooked food. The cooking process may involve conventional cooking methods (e.g., rice cooker, steaming, and boiling) or modern‐style techniques (e.g., microwave and autoclaving). There were previous studies reported that these cooking processes cause the great reduction of bioactive compounds and antioxidant activities in both pigmented and nonpigmented rice, comparing to those of uncooked rice (Chmiel, Saputro, Kusznierewicz, & Bartoszek, 2018; Surh & Koh, 2014). Among these processes, phenolic degradation of steam‐ and microwave‐cooked rice was found moderate extent. Furthermore, Chmiel et al. (2018) revealed that applying of microwave could retain higher phenolic composition of cooked rice during storage and reheating process in comparison with rice cooker and boiling methods.

The type of cooking method not only affects the changes to the bioactive compounds and other chemical properties of cooked rice, but they also impact on the physical properties as well (Rewthong, Soponronnarit, Taechapairoj, Tungtrakul, & Prachayawarakorn, 2011; Xu et al., 2019). Specifically, texture has been implied as one of the predominant parameters used to determine the quality of cooked rice and other food materials, due to its relationship with eating quality and consumer acceptance (Chen & Opara, 2013; Xu et al., 2019). Therefore, this research focused on the texture attribute and change in the bioactive compounds and antioxidant activity of cooked pigmented rice, comparing between microwave and steaming methods.

## 2 MATERIALS AND METHODS

### 2.1 Chemicals

Gallic acid monohydrate (ACS reagent, ≥98.0%), 2,2‐diphenyl‐1‐picrylhydrazyl (DPPH), and 2,4,6‐tris(2‐pyridyl)‐s‐triazine (TPTZ) were purchased from Sigma‐Aldrich. Trolox standard (HPLC grade) and Folin–Ciocalteu phenol reagent were purchased from Wako Pure Chemical Industries Ltd.

### 2.2 Rice sample and preparation

Selected Thai pigmented rice were examined: “Black” pigmented nonwaxy rice (cv. Hom Nin) was purchased from Pensook Company, Bangkok, Thailand; and “Red” pigmented nonwaxy rice (cv. Red Hommali) and “Purple” pigmented waxy rice (cv. Kum Luempua) were purchased from Smile Rice Brand, Chaiyaphum, Thailand. All pigmented rice cultivars were soaked in the ratio of 1:4.5 (rice:water, w/v) at 10°C for 19 hr. Soaked rice was cooked by using two different cooking methods; microwave and steaming. In the microwave method, rice with soaking water was transferred to a microwaveable ceramic pot and then placed in a microwave oven (MRO‐DF6, Hitachi) at 600 W for 12 min. Meanwhile, the soaking water was drained from soaked rice before applying the steaming method. In this method, rice was steamed using a household closed vessel stainless steel steam basket suspended above the amount of boiling water, for 25 min (black and red rice cultivars) and 40 min for purple rice cultivar, respectively. Both microwave‐cooked rice and steam‐cooked rice were compressed between two glass slides. No white core contained inside a cooked rice grain can be characterized as fully gelatinized and, thus, completely cooked. Completely cooked rice was wrapped in plastic film and incubated at 30°C for 30 min to equilibrate moisture throughout the grain; however, the incubation time was extended to 2 hr for texture analysis.

### 2.3 Cooked rice texture determination

After incubation for 2 hr, the 15 grains of cooked pigmented rice were analyzed for their firmness and adhesiveness, using a creep meter (RE2‐3305S, Yamaden Co. Ltd.). Each cooked grain was placed on a baseplate of the creep meter and compressed by a cylindrical probe (diameter of 30 mm) at a speed of 1 mm/s. The condition was set at 90% compression and 0.2 N of trigger force. The texture of cooked rice was measured within approximately 20 min, to avoid erroneous results due to moisture evaporation and alteration to the physical properties of the rice grains (Tamura et al., 2014).

### 2.4 Sample extraction

Cooked rice was freeze‐dried by using a freeze dryer (FDU‐1100, Tokyo Rikakikai Co. Ltd.) before extraction. Furthermore, uncooked rice grains and freeze‐dried samples were ground and passed through a 0.5‐mm sieve mesh. Two grams of sample was then extracted in 50 ml of 80% (v/v) methanol at ambient temperature for 6 hr under continuous shaking, using a modification of the method published by Abdel‐Aal, Young, and Rabalski (2006). The extracted sample was filtered through filter paper (Whatman no.1) to obtain the clear supernatant and then kept at 4°C for further analysis.

### 2.5 Total phenolic content (TPC)

According to the ISO14502‐1 (2005) method, 1 ml of diluted extract was mixed with 5 ml of 10% (v/v) Folin–Ciocalteu phenol reagent and 4 ml of 7.5% (w/v) Na_2_CO_3_, respectively. The mixture was left at ambient temperature for 1 hr before the absorbance was measured at 765 nm, using gallic acid monohydrate (ACS reagent, ≥98.0%) as a standard. The result was expressed as mg of gallic acid equivalents/100 g of dried sample.

### 2.6 Total anthocyanin content (TAC)

Determination of the TAC proceeded using a pH differential technique, as detailed by Giusti, Rodríguez‐Saona, and Wrolstad (1999). One part of the sample was mixed with four parts of two buffer reagents, respectively: KCl (0.025 M, pH 1.0) and NaOAc (0.4 M, pH 4.5). The mixtures were left to react at ambient temperature for 20 min, and then, the absorbance was measured at 520 and 700 nm, respectively, to remove the interference from the background. The result was expressed as mg of cyanidin‐3‐glucoside equivalents/L sample.

### 2.7 DPPH radical scavenging activity (DPPH)

This assay was performed by following the method of Molyneux (2004). Briefly, the mixture of diluted extract (50 µl) and 60 µM DPPH solution (1950 µl) was incubated in the dark for 30 min. The absorbance was measured at 517 nm, using methanol and Trolox as blank and standard, respectively. The result was expressed as µmol of Trolox equivalents/100 g of dried sample.

### 2.8 Ferric reducing antioxidant power (FRAP)

FRAP was evaluated using a slightly modified version of the procedure described by Benzie and Strain (1999). A 1.3‐mL aliquot of freshly prepared FRAP reagent (300 mM acetate buffer [pH 3.6], 10 mM TPTZ in 40 mM HCl, and 20 mM FeCl_3_ at 10:1:1 v/v/v ratio) was mixed with 200 µl of diluted extract, incubated at 37°C for 30 min, and the absorbance was measured at 517 nm, using ferrous sulfate (Fe[II]) as the standard. The result was expressed as µmol of ferrous sulfate equivalents/100 g of dried sample.

### 2.9 Statistical analysis

All data were evaluated by analysis of variance, followed by Duncan's multiple range test. The correlation between parameters was analyzed by Pearson's test (two‐tailed). All statistical analyses were performed using SPSS (version 20.0; IBM Corp.).

## 3 RESULTS AND DISCUSSION

### 3.1 Cooked rice firmness and adhesiveness

**Figure 1:**
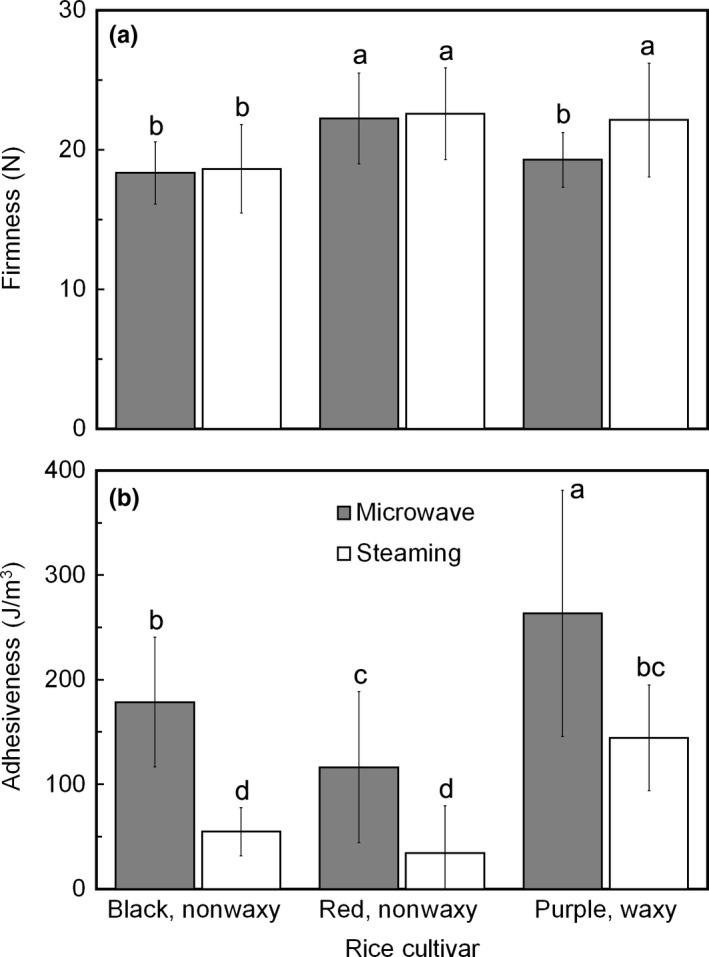
Texture property of cooked pigmented rice by microwave and steaming methods; (a) cooked rice firmness and (b) cooked rice adhesiveness. Different letters in the graph indicate a significant difference (*p* < .05) among samples (*n* = 15)

According to Figure 1a, firmness of microwave‐cooked rice and steam‐cooked rice was comparable. However, steam‐cooked purple rice showed higher firmness (*p* < .05) than microwave‐cooked purple rice, whereas this phenomenon did not occur in cooked black and red rice. The reason for the different rice firmness of cooked purple rice is not yet clear. The hypothesis is that purple rice cultivar leaches a different amount of amylopectin during cooking when compared with the other cultivars. Excess cooking water surrounding the rice grain during the microwave method could have facilitated high water absorption into the rice grain; increasing the water absorption during cooking and thereby generating the larger amount of leached starch (Tamura et al., 2014). This occurrence differs from the steaming method that required vaporized water for cooking. The leached starch composition is generally related to the amylose and amylopectin contents; short‐chain amylopectin has been reported as a major component of leached starch, as compared with long‐chain amylopectin and amylose (Ong & Blanshard, 1995). Furthermore, earlier research also insinuated that increased leaching of short‐chain amylopectin is responsible for decreasing the cooked rice firmness (Ong & Blanshard, 1995). Consequently, purple rice cultivar is waxy rice, due to its high proportion of amylopectin (Wani et al., 2012), and this might explain the significant decrease in firmness of microwave‐cooked purple rice. When comparing the cooked rice firmness among rice cultivars, regardless of cooking method, cooked red rice presented a higher firmness (22.25–22.59 N) than cooked purple rice (19.27–22.13 N) and cooked black rice (18.33–18.62 N), possibly because of the different structure and anatomy among cultivars (Ziegler et al., 2018).

Contrary to cooked rice firmness, adhesiveness of steam‐cooked rice was significantly lower than that of microwave‐cooked rice (Figure 1b). Differences in adhesiveness (i.e., stickiness) have been attributed to different amounts of water absorption into the rice grain during cooking; a large amount of water infiltration into rice grain results in enhanced adhesiveness (Tamura & Ogawa, 2012). A similar phenomenon could be applied to the leached short‐chain amylopectin during cooking, mentioned above. Leached amylopectin during cooking is believed to accumulate as a viscous coated layer to the outer surface of cooked rice, leading to increased adhesiveness (Yang et al., 2016). Meanwhile, this phenomenon could not be found in high‐amylose food materials (Syafutri, Pratama, Syaiful, & Faizal, 2016), which explains why waxy rice, such as cooked purple rice, demonstrated significantly higher adhesiveness than cooked nonwaxy rice (black and red rice), and the variation in the value between the microwave method and steaming method.

### 3.2 Bioactive compounds and antioxidant activities of pigmented rice grain

As seen in Table 1, the TPC and TAC in pigmented rice ranged from 637.68 to 914.37 mg/100 g dried sample and 0.6–35.61 mg/L sample, respectively. Purple rice cultivar contained the most TPC and TAC, followed by black rice and, lastly, red rice cultivars (*p* < .05). From this result, TAC is associated with TPC. The anthocyanins are a subclass of the flavonoids and are recognized as the major phenolic compounds in pigmented rice grains (Kushwaha, 2016), which exist mainly in dark‐colored grains rather than among pale‐colored grains (Yodmanee, Karrila, & Pakdeechanuan, 2011). Their color range can be characterized by the degree of hydroxylation in the B‐ring of their carbon skeleton; recognized as delphinidin‐, cyanidin‐ and pelargonidin‐glycoside forms by providing blue–purple, light purplish–red, and orange–red hues, respectively (Glover & Martin, 2012). It has already been confirmed that black–purple rice contains higher TPC and TAC than red rice, and cyanidin‐3‐glucoside was found to be the major anthocyanin detected in pigmented rice grains, with varying concentration among black–purple grains and red grains (Abdel‐Aal et al., 2006; Yao, Sang, Zhou, & Ren, 2010). Thus, since the purple rice cultivar exhibited dark reddish extract solutions, it could possess significantly greater amounts of bioactive compounds than black and red rice cultivars that displayed pink and yellowish–brown extract solutions, respectively.

**Table 1:** Bioactive compounds and antioxidant activities of uncooked rice

Rice cultivar	Total phenolic content (mg GAE/100 g dried sample)	Total anthocyanin content (mg cyanidin−3‐glucoside equivalents/L sample)	DPPH radical scavenging activity (µmol TE/100 g dried sample)	Ferric reducing antioxidant power (µmol Fe [II]/100 g dried sample)
Black, nonwaxy	807.63 ± 19.02^b^	12.14 ± 0.11^b^	3,347.38 ± 310.08^b^	11,868.71 ± 343.51^b^
Red, nonwaxy	637.68 ± 12.94^c^	0.60 ± 0.13^c^	2,699.92 ± 84.15^c^	9,005.03 ± 401.77^c^
Purple, waxy	914.37 ± 18.17^a^	35.61 ± 1.44^a^	3,971.70 ± 421.74^a^	15,933.47 ± 517.12^a^

Note

Different superscripts in the column indicate a significant difference (*p* < .05) among samples (mean ± *SD*; *n* = 3).

Abbreviations: GAE, gallic acid equivalents; TE, Trolox equivalents.

Phenolic compounds, including anthocyanins, are well‐recognized as powerful antioxidants that possess free radical scavenging activity, providing antioxidant defense and metal chelating properties (Dai & Mumper, 2010). In this study, the DPPH radical scavenging activity and FRAP assays, which are based on hydrogen atom transfer and electron transfer reactions, respectively, were applied for measuring the antioxidant activity, due to their simplicity and reproducibility (Dai & Mumper, 2010; Seo et al., 2013; Sompong et al., 2011). Purple rice cultivar significantly potent antioxidant DPPH and FRAP results (Table 1), which were approximately one‐ and 1.5‐fold more than those of black and red rice cultivars, respectively (*p* < .05), probably related to the TPC and TAC existing in the rice grains. Similarly, Walter et al. (2013) also reported a correlation between the antioxidant activity and the presence of phenolic compounds in rice extracts. Generally, the phenolic compounds act as a free radical acceptor and chain breaker by donating hydrogen atoms or electrons from their hydroxyl group to a free radical, as well as binding with a ferrous ion to reduce a free radical. The antioxidant capacity of the molecule depends on its number of hydroxyl groups and its structural characteristics (Dai & Mumper, 2010; Walter & Marchesan, 2011). Our result demonstrated a three‐ to fourfold greater FRAP activity than DPPH radical scavenging activity among the rice cultivars studied. It might indicate that phenolic compounds contained in pigmented rice grains tend to endow antioxidant effects by electron transfer through ferric complexes.

### 3.3 Changes to the bioactive compounds and their antioxidant activities in cooked pigmented rice

A dramatic decrease in the levels of the bioactive compounds and antioxidant activities of pigmented rice occurred after cooking (Table 2) when compared with their uncooked counterparts (Table 1). The reduction ratio was in the range of 2.9‐ to 5.1‐fold for TPC, 2.9‐ to 12.0‐fold for TAC, 1.6‐ to 6.0‐fold for DPPH antioxidant activity, and 2.0‐ to 8.5‐fold for FRAP, with the variation depending on the rice cultivars and cooking condition. Thermal processes, like cooking (Surh & Koh, 2014), drying (Norkaew et al., 2017), and pasteurization (Pérez‐Conesa et al., 2009), have been shown to result in deterioration of phenolic compounds and anthocyanins, which was associated with breakdown into other products as well as vaporization during heating. Normally, the water temperature is raised to near boiling temperature during cooking for complete rice starch gelatinization (Bhattacharya, 2011; Yu, Turner, Fitzgerald, Stokes, & Witt, 2017). As the temperature increases, the hydroxyl group of phenolic compounds would be destroyed. Then, phenolic compounds with high antioxidant activity could transform into smaller molecules or other products which may show low antioxidant activity (Saikia, Dutta, Saikia, & Mahanta, 2012; Sun, Bai, & Zhuang, 2014). A hydrophilic phenolic compound like anthocyanins is mainly found in pigmented rice and labile to the thermal process, light, and oxygen exposure (Patras et al., 2010; Surh & Koh, 2014). Herein, the high heating temperature might cause an opening of the pyrylium ring of anthocyanins, leading to cleavage of the glycoside linkage and formation of the chalcone structure (colorless form), indicative of the initial degradation step of anthocyanins (Patras et al., 2010). According to the previous study, anthocyanins could be further degraded by the transformation of chalcone structure into coumarin glucoside derivative with loss of B‐ring (Adams, 1973; Patras et al., 2010). Moreover, some studies also revealed that anthocyanins could convert into free phenolic acids (i.e., protocatechuic acid) during cooking, which was one of the major anthocyanin degradation products (Bhawamai et al., 2016; Yamuangmorn, Dell, & Prom‐u‐thai, 2018). In addition to anthocyanins, pigmented rice grain also possesses ferulic acid and p‐coumaric acid, which mostly presented as bound phenolic acids that mainly linked to cell walls as glycoside esters (Saikia et al., 2012). The cooking process could break down the cell‐matrix structure and facilitate the releasing of bound phenolics. The liberated bound phenolics might transform to free phenolics in which readily decomposed under high heating temperature (Saikia et al., 2012; Scaglioni, de Souza, Schmidt, & Badiale‐Furlong, 2014; Zeng, Liu, Luo, Chen, & Gong, 2016), as well as generating some chemical reaction with macromolecules or other compounds and led to change in content and structure of phenolics (Shahidi & Yeo, 2016). Consequently, there is a possibility that these phenolics, particularly hydrophobic ones, could form an inclusion complex with starch components, especially amylose, during cooking, resulting in the reduction of phenolic compounds (Surh & Koh, 2014). Additionally, a chemical reaction between proteins and phenolics via irreversible covalent bonds might induce the formation of nonextractable fractions, causing the reduction of phenolic compounds (Ozdal, Capanoglu, & Altay, 2013; Shahidi & Yeo, 2016). Nonetheless, the impact of cooking on the degradation mechanism of anthocyanins and phenolic acids needs to identify and quantify in further study. The reduction of TPC and TAC during cooking would also decrease the antioxidant activities of cooked pigmented rice. Our result was consistent with the finding of Gong et al. (2017) which indicated that phenolic compounds were responsible for the antioxidant capacity of rice grain. These observations confirm that the cooking process is one of the crucial factors influencing the degradation of bioactive compounds and antioxidant activities in pigmented grains.

**Table 2:** Bioactive compounds and antioxidant activities of cooked rice

Rice cultivar	Cooking condition	Total phenolic content (mg GAE/100 g dried sample)	Total anthocyanin content (mg cyanidin−3‐glucoside equivalents/L sample)	DPPH radical scavenging activity (µmol TE/100 g dried sample)	Ferric reducing antioxidant power (µmol Fe [II]/100 g dried sample)
Black, nonwaxy	Microwave	234.91 ± 6.48^b^	3.06 ± 0.15^c^	891.22 ± 15.41^b^	3,233.36 ± 301.34^b^
	Steaming	185.56 ± 6.20^c^	1.55 ± 0.04^d^	678.43 ± 8.54^c^	2,209.89 ± 102.90^c^
Red, nonwaxy	Microwave	154.43 ± 2.97^d^	0.08 ± 0.05^e^	487.10 ± 37.46^cd^	1,132.35 ± 98.77^d^
	Steaming	124.61 ± 1.30^e^	0.05 ± 0.01^e^	446.64 ± 42.12^d^	1,055.71 ± 18.94^d^
Purple, waxy	Microwave	455.34 ± 27.06^a^	12.17 ± 0.29^a^	2,409.46 ± 231.09^a^	7,972.40 ± 579.82^a^
	Steaming	231.83 ± 21.67^b^	3.85 ± 0.31^b^	954.04 ± 154.14^b^	3,241.23 ± 167.72^b^

Note

Different superscripts in the column indicate a significant difference (*p* < .05) among samples (mean ± *SD*; *n* = 3).

Abbreviations: GAE, gallic acid equivalents; TE, Trolox equivalents.

Furthermore, the type of cooking process influenced the bioactive compounds and antioxidant activities of cooked pigmented rice. The TPC, TAC, DPPH, and FRAP values of steam‐cooked rice were remarkably degraded relative to microwave‐cooked rice (*p* < .05) (Table 2). This outcome could be expected given the observed leaching of hydrophilic phenolic compounds from the aleurone layer of the rice grain into the soaking water (Table 3). The leached anthocyanins and other water‐soluble phenolic compounds in the soaking water accounted for 18%–51% and 6%–22%, respectively, with the varying concentrations depending on the pigmented rice cultivar. As a result, the soaking process could be the initial step for loss of bioactive components in pigmented grain due to migration of soaking water toward embryo regions and then infiltrated into the aleurone layer and other internal tissues (Hong et al., 2009). This could generate expansion of cell‐matrix structure and cracks inside soaked rice, resulting in anthocyanins and other hydrophilic phenolic substances, those mainly located in aleurone layer, could be easily liberated into soaking water (Hong et al., 2009; Yamuangmorn et al., 2018). Earlier publications have noted a similar finding when investigating the impact of the soaking process on the leaching of phenolic compounds from legumes and their antioxidant activities (Siah et al., 2014; Xu & Chang, 2008). They demonstrated that approximately 40%–68% of phenolic compounds in legumes were leached out to soaking and cooking water, causing a reduction in bioactive substances and antioxidant capacity of legumes. Typically, soaking water is discarded before the steaming process that is standard practice across Asian households, indicating that a limited quantity of bioactive components in first soaked grain. Likewise, the study of Yamuangmorn et al. (2018) affirmed that the presoaking process was a notable influence on the deterioration of water‐soluble phenolic compounds (i.e., anthocyanins) in pigmented rice, which was found 16%–65% loss after soaking process with varying among rice genotypes. Apart from leaching of bioactive compounds into soaking water, higher heating rate and vapor water during the steaming process have been indicated to cause rapid evaporation of free water in the rice grains as well as generate porous structure (Piyawanitpong, Therdthai, & Ratphitagsanti, 2018). This could probably lead to liberate phenolic compounds that linked to the cell‐matrix structure and subsequently interact with the rice starch component, as mentioned above. This might connect to the reduction of bioactive compounds concentration and antioxidant activities of steam‐cooked rice.

**Table 3:** Bioactive compounds and antioxidant activities of rice's soaking water

Rice cultivar	Total phenolic content (mg GAE/100 g dried sample)	Total anthocyanin content (mg cyanidin−3‐glucoside equivalents/L sample)	DPPH radical scavenging activity (µmol TE/100 g dried sample)	Ferric reducing antioxidant power (µmol Fe [II]/100 g dried sample)
Black, nonwaxy	93.36 ± 3.28^b^	3.43 ± 0.84^b^	330.31 ± 35.74^b^	1,366.54 ± 100.40^b^
Red, nonwaxy	36.18 ± 2.86^c^	0.11 ± 0.04^c^	179.00 ± 13.61^c^	564.29 ± 29.74^c^
Purple, waxy	199.46 ± 1.46^a^	18.19 ± 1.43^a^	1,219.40 ± 78.98^a^	4,358.32 ± 347.29^a^

Note

Different superscripts in the column indicate a significant difference (*p* < .05) among samples (mean ± *SD*; *n* = 3).

Abbreviations: GAE, gallic acid equivalents; TE, Trolox equivalents.

Extending the duration of cooking has also been identified as a parameter influencing the loss of bioactive compounds and their antioxidant ability (Bhawamai et al., 2016; Chuah et al., 2008). The study of Bhawamai et al. (2016) regarding impact of thermal cooking on profile of phenolic compounds in black rice revealed that applying shorter cooking time could possibly retain high anthocyanin content in black rice. In this context, the shorter cooking time of microwave cooking method (12 min) might justify more bioactive compounds and, consequently a higher antioxidant activity of cooked pigmented rice than that of the steam cooking approach (25–40 min of cooking). In addition, microwave cooking allows complete absorption of water into rice grains, in which this phenomenon has been confirmed to retain more phenolic compounds and antioxidant capacity in cooked pigmented rice (Zaupa et al., 2015). Nevertheless, we found that the type of cultivar and bran color also affected these attributes. As a result, the phenolic compounds and their antioxidant activities mostly existed in cooked purple rice, followed by cooked black rice and, lastly, cooked red rice (*p* < .05).

### 3.4 Correlation of TPC, TAC, DPPH, and FRAP

Pearson's correlation analysis (Table 4) evidenced a strong positive correlation between TPC and TAC, confirming that anthocyanins were the predominant phenolic compound contained in pigmented rice grain. This result corroborated with an earlier study, as well (Surh & Koh, 2014). Additionally, the bioactive compounds (TPC and TAC) had a high positive correlation with the DPPH and FRAP antioxidant activities, with the FRAP value showing a slightly higher positive relationship with TPC and TAC. This suggested that the phenolic compounds contributed to most of the antioxidant potential and might preferentially act as reducing agents rather than radical scavengers.

**Table 4:** Pearson correlation between total phenolic content (TPC), total anthocyanin content (TAC), DPPH radical scavenging activity (DPPH), and ferric reducing antioxidant power (FRAP) of all pigmented rice cultivars

	TPC	TAC	DPPH	FRAP
TPC	1.000	0.772*	0.984*	0.987*
TAC		1.000	0.782*	0.846*
DPPH			1.000	0.983*
FRAP				1.000

* Correlation is significant at *p* < .01 (two‐tailed).

## 4 CONCLUSION

Texture is a key parameter determining the cooked rice quality. Our result showed that the firmness of microwave‐cooked rice was comparable to steam‐cooked rice, despite microwave cooking promoting a higher adhesiveness. This study also affirmed the cooking process as a critical factor affecting the deterioration of TPC and TAC and, consequently, it was responsible for decreasing the antioxidant activities (DPPH and FRAP) of cooked pigmented rice. Shorter cooking time and cooking pattern of microwave could better maintain the bioactive compounds and the antioxidant ability of cooked pigmented rice rather than the steaming method. Hence, microwave cooking can be considered a recommended cooking method for consumers and manufacturers for preserving the antioxidant components in cooked rice and reducing the cooking time. Further studies are required to identify the optimal microwave conditions.

## CONFLICT OF INTEREST

The authors declare no conflict of interest. The project was funded by The Tojuro lijima Foundation for Food Science and Technology, but they had no role in study design, data collection, or analysis. The authors alone are responsible for the content and writing of the paper.

## Ethics Statements

Our research did not include any human subjects and animal experiments; however, this study was designed to conform to the Declaration of Helsinki, US, and/or European Medicines Agency Guidelines for human subjects.
